# Assessment of intention to use postpartum intrauterine contraceptive device and associated factors among pregnant women attending antenatal clinics in ambo town public health institutions, Ethiopia, 2018

**DOI:** 10.1186/s40834-021-00152-x

**Published:** 2021-04-01

**Authors:** Gurmessa Daba, Jembere Tesfaye Deressa, Workinesh Sinishaw

**Affiliations:** 1 Ambo University College of Medicine and Health Science, Ambo, Ethiopia; 2 Addis Ababa University College of Health Science, School of Nursing & Midwifery, Addis Ababa, Ethiopia

**Keywords:** Antenatal care, Intention, Postpartum intrauterine contraceptive device, Pregnancy

## Abstract

**Background:**

Maternal mortality tragedy is the issue of both developed and developing countries, especially sub-Saharan Africa including Ethiopia, which is due to poor quality of maternal health care services. Therefore family planning especially the use of Postpartum intrauterine contraceptive devices can tackle unintended pregnancy and maternal death. However,the intention to use PPIUCD and the use of IUCD in general is not well practiced in Ethiopia according to evidences of the literatures. For this reason, many mothers are exposed to unintended pregnancy and pregnancy related complications. The main purpose of the study was to assess the intention to use Post-partum intra uterine contraceptive devices and its associated factors among pregnant women attending Antenatal Clinics in Ambo Town Public Health Institutions, Ethiopia, 2018.

**Methods:**

A facility-based cross-sectional study design was conducted from March – April 2018 to assess the intention of pregnant women about Postpartum intrauterine contraceptive devices and associated factors in 422 pregnant women. Study subjects were selected using systematic random sampling. Data were collected by structured questionnaire, entered into a computer using Epi-info 3.5.4 statistical program, and exported to SPSS version 20 for analysis. A Logistic regression model was used to predict the intention of pregnant women about Postpartum intrauterine contraceptive devices and associated factors. Lastly, a significant statistical association was tested using 95% confidence interval (CI) and *p* value (*p* < 0.05).

**Results:**

The response rate was 417 (98.3%) and this study showed that 145(34.9%) of pregnant women intended to use Postpartum intrauterine contraceptive device. Age of pregnant women [AOR = 8.348(CI: 3.602–19.347], educational level [AOR = 3.249(1.057–9.985)], occupational status [AOR = 4.101(CI: 1.788–9.405)], monthly income [AOR = 3.175(CI: 1.423–7.082)] and knowledge [A0R = 5.408(2.994–9.767)] have shown significant associations with intention to use Postpartum intrauterine contraceptive devices.

**Conclusions:**

The study found that 34.9% women intended to use PPIUCD. Maternal age, maternal educational level, occupational status, monthly income and knowledge were significantly associated with pregnant women intention to use PPIUCD.

## Introduction

An IUCD is a small, “T-shaped” intrauterine contraceptive devices, which is placed in a woman’s uterus. It is also known as the IUD, loop or coil. Postpartum IUCD is an intrauterine contraceptive device which is inserted during the postpartum period (up to 48 hrs after birth, optimally within10 min of delivery of the placenta). IUCDs are prepared of flexible plastic with thin copper wire coating. It has one or two soft threads on the last part. These thin threads hang through the opening at the entrance of the uterus into the top of the vagina [1].Copper-bearing IUDs come in a variety of designs the IUD recommended by WHO for bulk obtaining is the TCu380A it works primarily by causing chemical changes that prevent fertilization. Studies show that the copper IUD effectively interrupts the reproductive process before implantation and pregnancy and it does not act by initiating an abortion, as has sometimes been suggested [2]. Renewed interest in the intrauterine device (IUD), a highly effective, long-acting reversible contraceptive (LARC) that is safe for breastfeeding women, can be inserted in a matter of minutes by a trained provider, and do not require an additional facility visit when inserted during the childbirth stay has encouraged some programs to add postpartum IUD services to their PPFP options [3–5]. Ethiopia in collaboration with Maternal and Child Health Integrated Program (MCHIP) started the PPIUCD program in 2012, PPIUCD services were initiated as one FP option for postpartum clients [6, 7].

The most successful PPFP programs will focus on providing PPFP counseling to women at every opportunity. In low-income countries, increasing emphasis on antenatal care and childbirth in a health care facility has created an opportunity to counsel women about family planning. The health benefits of contraception and birth spacing for women and their infants are remarkable and the woman is not pregnant at the time of insertion and is protected against pregnancy prior to resuming sexual activity [3].

Appropriate times for IUCD insertion in the postpartum periods include the post placental IUCD insertion, the immediate postpartum IUCD insertion and the trance cesarean IUCD insertion. The post placental IUCD insertion is done within 10 minutes after expulsion of the placenta, following a vaginal delivery. The immediate postpartum IUCD insertion is done after the post.

placental period, but within 48 hrs of delivery and the trance cesarean IUCD insertion is when the insertion takes place following a cesarean delivery, before the uterus incision is sutured [7, 8]. These periods the cervix is open and limp and an IUCD can easily be placed high in the fundus, either manually or using forceps. Furthermore it continues to be possible to insert an IUCD with an instrument for up to 48 h postpartum [8]. After birth, uterine contractions expel retained placental tissues and blood clots and may have a similar effect on any foreign body introduced into the uterus. IUCDs inserted postplacentally have a much lower expulsion risk than those inserted later in the postpartum period, although the expulsion is still higher than for interval insertions. However, the benefits of providing highly effective contraception immediately after delivery often outweigh the disadvantage of the higher postpartum expulsion rates [8]. Two reasons informed the decision to focus on contraceptive intention. First, intention has been posited in many theories of behavior change as the most proximate determinant of actual behavior. Also, many studies have found intention to be a very strong determinant of behavior. Second, in the absence of longitudinal data, the focus on the association of ideation with intention to use as opposed to actual contraceptive use allows us to better address possible reverse causality inherent in cross-sectional data. Behavioral intentions (an indication of an individual’s readiness to perform a given behavior it is assumed to be an immediate antecedent of behavior) relate to how people see themselves in the future [9]. The information derived from this study would provide directions for both governmental and non-governmental bodies to implementing successful strategies that are effective in promoting use of PPIUCD in maternal health service utilization areas like ANC and FP which eventually leads to the improvement of CPR and health of the women. So, intention to use PPIUCD which is effective, safe, reversible and long acting method of contraception is going to be crucial in meeting the above needs because there is a limited study conducted to assess intention to use PPIUCD use and factors affecting intention to use PPIUCD in Ethiopia.

## Methods

### Study design and study period

Institution based cross sectional study design was used to collect data from pregnant women attending antenatal clinic from March 1, 2018- April 30, 2018.

### Source population

All women who are in the reproductive age group (15–49 years old) and living in the Ambo town.

### Study subjects

From the source population women those who are pregnant and attending ANC Clinics in Ambo town during data collection period.

### Inclusion and exclusion criteria

All reproductive and pregnant women aged 15–49 years were included to the study but seriously sick and those who unable to respond like laboring mothers were excluded.

### Sample size determination

The sample size was calculated by using single population proportion formula based on the following assumptions: Since there is no appropriate population value to calculate n from the reviewed literature and it was not only done on pregnant women the proportion was taken at 50%. Significant level at α = 0.05, at 95% confidence interval, Margin of error is 5 and 10% nonresponsive rate, the sample size is calculated by the following formula:

*n* = (Zα/2)2 P (1-p) d 2 *n* = (1.96)2 (0.5) (0.5) = 384 (0.05)2 Where: *n* = the required Sample size *p* = prevalence of intention to use PPIUCD (50% or *P* = 0.5) Z = the value of the standard normal curve score corresponding to the given Confidence interval 1.96 d = the permissible Margin of error (the required precision) = 5%.

By adding 10% of non-response rate, total of 422 pregnant women will be recruited as study units among pregnant women who attended ANC follow up at health facilities in Ambo town during study period.

### Sampling procedure

In this study, all public health institutions which provide ANC service were selected. Based on these, three public health institutions, (one hospital and two health centers) was included. The total population for these health institutions (their average monthly ANC flows of previous, at least for 2 months is estimated that 928, (321 for Ambo Hospital, 396 for Ambo health center and 211 for Awaro health center) so final sample size (422) is obtained by proportionally allocating to these selected health facilities by considering their monthly ANC flows. Lastly, subjects (pregnant mother) were taken by systematic Random sampling. Based on assessment of each health facility monthly load, by systematic random sampling technique (i.e. Kth = N / sample size= > 928 /422 ≈ 2 which means K th = 2), thus every 2nd pregnant women who attended this facility was recruited as study units in each health facilities until the total sample size for this study is obtained.

### Data quality control/ assurance

The data collection instrument was pre-tested for its relevance and clarity to address the research problems appropriately and corrected prior to the actual data collection period. Pretest was done at another health center, before conducting the major study on 5% of the sample to check consistency of questionnaire. The authors checked for completeness and consistencies of questionnaires filled by the data collector and ensured the quality of the data.

### Operational definition

#### PPIUCD

An intrauterine contraceptive device that can be inserted post placentally, intra cesarean and within 48 hrs of delivery.

#### Intention to use PPIUCD

pregnant women who responded “yes” or “no”to use PPIUCD method after delivery within 48 hrs.

### Awareness of PPIUCD:simply information responded by the participants whether the participants ever heard of PPIUCD and the source of the information

#### Knowledge on PPIUCD

Pregnant women’s awareness of the existence of PPIUCD, its importance and effectiveness.

PPIUCD knowledge was measured by the total number of correct answers to 10 items on knowledge with a minimum score of 0 and maximum of 10. It was categorized based on the percent of knowledge of the distinct characteristics of PPIUCD as: “high” - those who scored 80% and above, “moderate” those who scored 60 - 79% and “low” those who scored less than 60%.

#### Attitude on PPIUCD

The likert scale with scores ranging from 1=strongly disagree to 5=strongly agree was used. To measure the attitude of the pregnant women’s towards intention to use PPIUCD two categories were assigned. Data was checked for normal distribution normally distributed so, the mean was used to measure attitude.

Positive Attitude - those who scored above the mean on attitude items and Negative Attitude - those who scored the mean or below mean to attitude items.

### Data entry and analysis procedure

After data collection, the questionnaire was checked for completeness. The collected data was entered in to Epi-info version 3.5.4 and exported to Statistical Package for Social Science (SPSS) version 20 for analysis. To identify the existence of association between the selected dependent and independent variables, bivariate logistic regression with 95% CI at *p*-value < 0.2 and multivariate logistic regression modelling with 95% CI at *p*-value < 0.05 was used. For all of statistical test used in this study, descriptive statistics such as frequency distribution and measure of central tendency and variability (mean and standard deviation) was computed to describe variables of the study. A confidence interval of 95% was used and thus a *p*-value < 0.05 was considered significant.

## Results

### Socio-demographic characteristics of pregnant women intention to use post-partum IUCD

The complete response rate of this study was 415(98.3%). Majority of the study participants were 115 (27.7%) in the age range of greater than 27 years. The mean age of the respondent was 24.23 ± 3.91 SD years. One hundred fifty three (36.9%) of the participants have completed grade 9–12, followed by one hundred thirty four (32.3%) those who completed grade 1–8. One hundred forty two (34.2%) were getting monthly income of less than 1000 ETB (Ethiopian Birr).

### Reproductive characteristics of the participants

The mean age at marriage was 19.23 (±2.45 SD) years. Majority, 286 (68.9%), of the women had ever given birth and more than three-fourth, 298 (71.8), had less than 2 ever born children. The mean number of alive and more wanted children in life was 1.56 (±1.48 SD) and 2.3 (±1.15 SD) respectively. Most, 336 (81%), women discussed about FP with their husbands and the number of children was decided both by the mother and father in more than three-quarters 344 (82.9%) of the families (Table 1).

**Table 1:** Reproductive characteristics of intention to use post-partum IUCD in Ambo town public health institution, Ethiopia, May 2018. (*n* = 417)

Variables	Number	Percent
Age at first marriage		
≤ 18 years	154	37.1
19-20 years	184	44.3
≥ 21 years	77	18.6
Ever give birth		
Yes	286	68.9
No	129	31.1
Number of birth		
≤ 2	298	71.8
3+	117	28.2
Number of alive children		
≤ 2	228	75.6
3+	101	24.4
Number of children want to have in life		
≤ 3	398	79.7
> 4	84	20.2
Want to have child within two years		
Yes	49	11.8
No	366	88.2
Discuss on family planning methods with partner		
Yes	336	81.0
No	79	19.0
Decision on the number of children want to have		
Husband	17	4.1
Wife	54	13.0
Both	344	82.9
Ever used family planning methods previously		
Yes	348	83.9
No	67	16.1
Method you used previously		
Natural family planning	3	0.7
Pills	114	27.5
Injectable	253	61.0
Implanon	119	28.7
IUCD	11	2.7
Condom	12	2.9

### Participant’s awareness about PPIUD

One hundred seventy six (42.6%) of the participants have good awareness about PPIUCD. Majority of the study participants, 121(29.2%), had awareness about PPIUD from a Mass media where as only 18(4.3) of the women heard about PPIUD from husband. There were 60(14.5%) women who heard about PPIUD from a family member/friend, 104(25.1%) heard from Health professionals.

### Knowledge of pregnant women to use post-partum intrauterine contraceptive

This study showed 205 (49%) of the respondents were found to have good knowledge, while210 (51%) of the respondents was not on intention to use postpartum IUCD. One hundred sixty (38.6%) of the participants know that PPIUD can prevent pregnancies for more than 10 years. One hundred forty-five (34.9%) of the participants know PPIUD is not appropriate for females at high risk of getting STIs. While only 100 (24.1%) know PPIUD has no interference with sexual intercourse or desire. Majority of the respondents know PPIUD can be removed at any time they want 174 (41.9%) (Table 2).

**Table 2:** Knowledge of pregnant women about PPIUCD in Ambo public health institution, Ambo, Ethiopia, May 2018. (*n*=417)

Knowledge About PPIUD			LEVEL OF KNOWLEDGE				Correct	Percent
							Response	Correct
			Yes		No			
			no	%	No	%		
	PPIUD can prevent pregnancies for more		160	38.6	54	13	160	38.6
than 10 years.								
PPIUD is not appropriate for females at high risk of getting STIs.			145	34.9	69	16.6	145	34.9
PPIUD has no interference with sexual intercourse or desire.			100	24.1	100	24.1	100	24.1
PPIUD is immediately reversible (become pregnant quickly when removed).			151	36.4	63	15.2	151	36.4
PPIUD does not cause cancer.			114	27.5	99	23.9	114	27.5
Breast feeding mothers can use PPIUD.			153	36.9	61	14.7	153	36.9
PPIUD may cause changes in bleeding pattern			121	29.2	92	22.2	121	29.2
PPIUD can be used by HIV positive patients doing well on treatment.			100	24.1	114	27.5	100	24.1
PPIUD is inserted free of charge in Ethiopia.			165	39.8	48	11.6	165	39.8
PPIUD can be removed at any time			174	41.9	36	8.7	174	41.9

### Intention of pregnant women to use PPIUCD

**Fig. 1:**
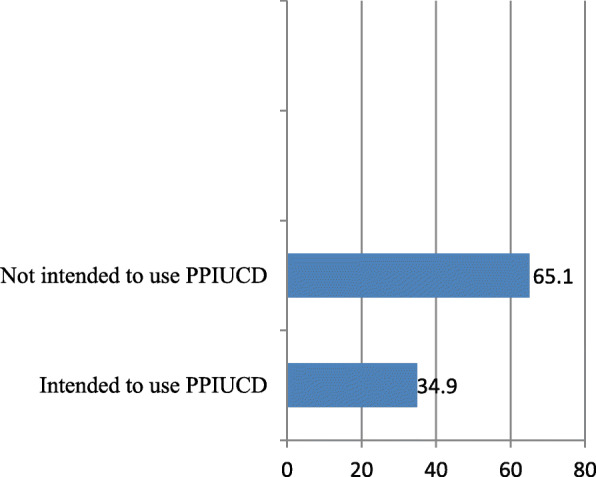
Intention to use Post-partum intra uterine contraceptive method among pregnant women attending antenatal clinics in Ambo town public health institutions, Ethiopia, 2018

**Fig. 2:**
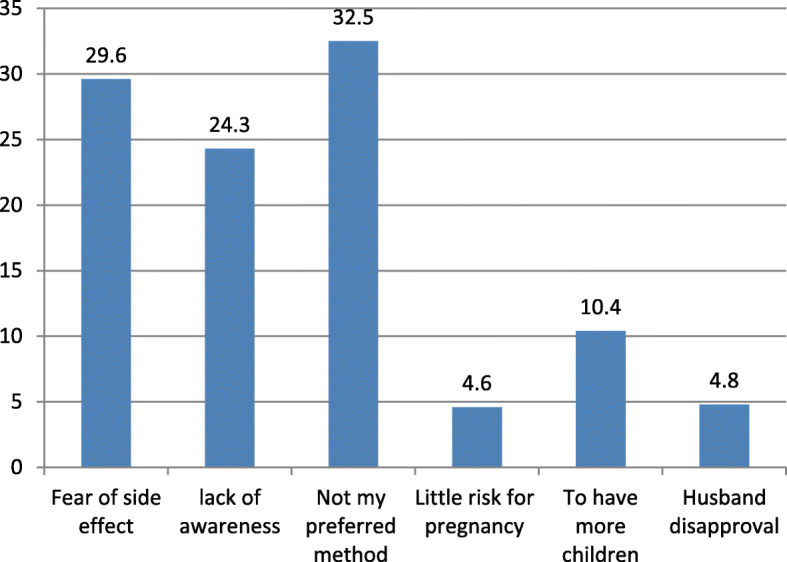
The reasons not preferring PPIUD contraceptive method among pregnant women attending antenatal clinics in Ambo town public health institutions, Ambo, Ethiopia, 2018

This study showed that 145(34.9%) of pregnant women intended to use PPIUCD while more than half of the study participant 270(65.1%) was not intended to use PPIUCD (Fig. 1). The reason not intended to use postpartum intrauterine device showed (Fig. 2).

### Participant’s attitude towards PPIUD

**Fig. 3:**
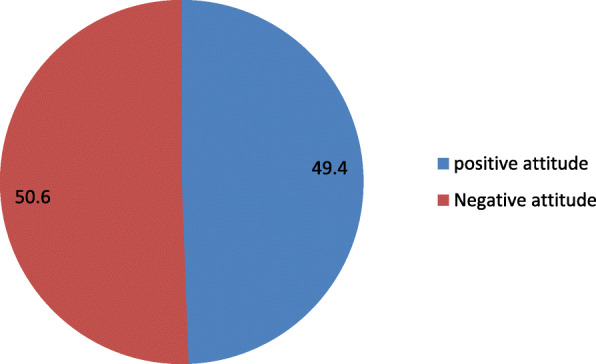
Pie chart showing Clients’ Attitudes towards PPIUCD contraceptive method among pregnant women attending antenatal clinics in Ambo town public health institutions, Ambo, Ethiopia, 2018

In order to ascertain the respondent’s level of attitude on pregnant women intention to use post-partum IUCD, the respondents were asked to reflect their opinion on a serious of questions concerning intention to use postpartum IUCD. Attitudes towards PPIUD were summarized through five issues related to: Insertion of PPIUCD inside the uterus does not lead to lose of privacy, Using PPIUCD does not restrict normal activities, PPIUCD doesn’t move through the body after insertion, PPIUCD does not interfere with sexual intercourse and PPIUCD can harm a woman’s womb. There was high disagreement about PPIUD insertion inside the uterus does not lead to lose privacy 223 (53.7%). Majority 260(62.7%) of the participants agree that using PPIUCD does not restrict normal activities. Also 254(61.2%) believe that PPIUCD does not move through the body after insertion and 147 (35.4%) felt PPIUCD can interfere with sexual intercourse. Majority 234(56.3%) of the participants disagree with PPIUCD can harm a woman’s womb. Below Fig. 3 presented overall positive and negative attitude of pregnant women about intention to use postpartum intrauterine contraceptive.

### Bivariate and multivariate logistic regression analysis of pregnant women intention to use PPIUCD and its explanatory variables

Binary Logistic regression was performed to assess the association of each independent variable with intention to use PPIUCD. After controlling the confounding factors, the multivariate revealed that the following factors have association with intention to use PPIUCD: Respondents age, educational status, respondent’s occupational status, respondent’s income and respondent’s knowledge (Table 3).

**Table 3:** Bivariate and multivariate logistic regression analysis of pregnant women’s intention to use post-partum intrauterine contraceptive device. (*n* = 417)

Variables	Intended To Use PPIUCD		COR(95%CI)	AOR(95%CI)	*p*-value
	Yes	No			
Age					
≥27	71(17.1%)	44(10.6%)	7.973(4.193–15.163)*	8.348(3.602–19.347)**	0.001
25–26	44(10.6%)	61(14.7%)	3.564(1.861–6.825)*	2.325(1.013–5.333)**	0.010
23–24	13(3.1%)	81(19.5%)	0.793(.362–1.737)	0.561(0.211–1.493)	
≤ 22	17(4.1%	84(20.2%)	1	1	
Educational status					
College and above	47(11.3%)	30(7.2%)	5.092(2.305–11.249)*	3.249(1.057–9.985)**	0.040
Secondary (9–12)	69(16.6%)	84(20.2%)	2.670(1.298–5.491)*	1.650(0.612–4.450)	
Primary (1–8)	17(4.1%)	117(28.2%)	0.472(.207–1.075)	0.375(0.129–1.092)	
No formal education	12(2.9%)	39(9.4%)	1	1	
Occupation					
Goven‟t employee	48(11.6%)	25(6%)	8.704(4.661–16.253)*	4.101(1.788–9.405)**	0.001
Private employee	53(12.6%)	74(17.8%)	3.431(2.027–5.806)*	2.772(1.395–5.506)**	0.004
Student	5(1.2%)	14(3.4%)	1.295(.481–3.485)	2.748(0.536–14.083)	
Daily laborer	6(1.4%)	21(5.1%)	1.619(.542–4.840)	0.639(0.179–2.285)	
House wife	30(7.2%)	136(32.8%)	1	1	
Personal income					
> 2501	51(12.3%)	45(10.8%)	5.304(2.942–9.561)*	3.175(1.423–7.082)**	0.005
1501–2500	53(12.8%)	77(18.6%)	3.221(1.848–5.616)*	1.970(0.935–4.150)	
1001–1500	16(3.9%)	31(7.5%)	2.415(1.150–5.073)	1.694(0.649–4.426)	
< 1000	25(6%)	117(28.2%)	1	1	
High knowledge	115(27.7%)	90(21.7%)	16.76(4.961–56.63)*	10.338(2.59–41.49)**	.001
Moderate knowledge	30(7.2%)	180(43.4%)	1	1	
Low knowledge	38(9.2%)	195(47.0%)	.223(.137–.362)	.288(.158–.528)	.001

*statistically significant at *p*<0.2 in bivariate

**statistically significant at *p*<0.05 in multivariable logistic regression

The result revealed that age of the respondent was among the variables that were found to be associated with pregnant women intention to use PPIUCD. Pregnant women’s who were in the age group of greater than 27 were eight times more likely to intended to use PPIUCD than those who were in the age group of less than 22 (AOR = 8.348, 95% CI: (3.602–19.347) (*P* < 0.05).

The other variables that were found to have association were the participant’s educational level. Pregnant women who were college and above of education were three times more often intend to use PPIUCD when compared with those who are no formal education. (AOR = 3.249, 95%(CI: (1.057–9.985) (*P* < 0.05).

Type of job was also found to be among the factors affecting pregnant women intention to use PPIUCD. Pregnant women who were government employee are four times more likely to intention to use PPIUCD than housewife (AOR = 4.101, 95% (CI: (1.788–9.405) (*P* < 0.05).

Pregnant women monthly income was also significantly influenced intention to use PPIUCD. Pregnant women whose monthly income of greater than two thousand five hundred (> 2500) were three times more likely intend to use PPIUCD than whose monthly income of less than one thousand (< 1000) (AOR = 3.17,95% (CI:(1.423–7.082) (*P* < 0.05).

Furthermore, knowledge of pregnant women was also found to affect the outcome variable. Respondents who had high knowledge about PPIUCD were about ten times more likely to intend to use PPIUCD than those with m knowledge (AOR = 10.338, 95% (CI: 2.59–41.49) (*P* < 0.001).

## Discussion

It was observed that the level of intention to use PPIUD among clients was low. The most appealing reason not intended to use PPIUCD was not their preferred method and fear of side effect.

This study showed the level of women intention to use PPIUCD was 34.9%, this finding was in line with findings done in Wolaita zone, (38%), Southern Ethiopia [10]. However,the present finding is inconsistent with the study done in North West Ethiopia in Adgrat town, western Ethiopia Nekemt town and Debiremarkos town were (44.6%),(47.9%) and (45.9%) of women intention to use PPIUCD respectively [11–13]. Those levels of variation may be attributed due to socio-demographic characteristic variation among study areas.

In this study, intention to use PPIUCD was significantly higher among older participants (AOR = 8.348(3.602–19.347) (*P* = 0.001) than younger participants. This finding was similar to other studies conducted in Kenya [14]. The present study is different from study done in Kenya, and Nigeria [9, 15]. This inconsistency is may be due to the variation of respondents‟ socio demographic characteristics and may be due to time gap of the study and sample size. Educational status was found to be associated with women intention to use PPIUCD in which those who were diploma and above are three times more likely to involve than with no formal education (AOR = 3.249,95%(CI:(1.057–9.985)(*P* < 0.05). This was comparable with other studies conducted in EDHS 2016 contraceptive uses increased dramatically with increase in level of education with at least 51% of women with secondary education and higher are using family planning, compared to 31% of women who had no education, Jimma, Wolaita in which women who attained secondary and higher level of education were found to be 2 and 3 times more likely to have the intention to use LAPMs compared to women who had no education, respectively and Nekemt women who had secondary school education and above were 1.82 times more likely to have intention to use LAPMs compared to those who had primary school education [10, 16–18]. This might be due to the fact that educated women might discuss more sensitive issues openly and freely they become closer and familiarized to each other. In addition women with some basic level of education can better understand the advantages and complications associated with intention to use PPIUCD.

Finding in this study indicated that pregnant mothers who were employed or government employee and private employee (AOR = 4.195% CI (1.78–9.4) *p* < 0.05and AOR = 2.7 95% CI (1.39–5.56) *p* < 0.05times more intended to use PPIUCD than daily laborer respectively. This study was consistent with the study conducted in western Ethiopia Nekemt town showed that government employed women were 2.6 times more likely express future intention to use LAPMs of contraceptive than women in other occupation [9] but inconsistent with the study done in Adgrat town [19]. This inconsistency is may be due to the variation of respondents‟ socio demographic characteristics and may be due to sample size.

Furthermore, women intention to use PPIUCD was found to be significantly associated with personal income in which pregnant women whose monthly income was high were three times more likely involve than whose monthly income was low (AOR = 3.17,95% CI:1.423–7.082)(*p* < 0.05). This finding was similar to EDHS 2016 Use of modern contraception increases sharply with wealth, ranging from 20% for women in the lowest wealth quintile to 47% for women in the highest wealth quintile [11] and also similar to study conducted in Nigeria [9]. This could be due to exposure of women for reproductive related information and indeed employed women have better decision making power on fertility issues.

Furthermore women intention to use PPIUCD was significantly higher among participants who had good knowledge on intention to use PPIUCD (A0R = 5.4, 95% (CI: 2.99–9.76) (*P* < 0.05) than those with poor knowledge. This finding was similar to other studies conducted in, Kenya‟s city‟s slums, Adgrat town and Mekelle town mothers who had high knowledge were 8 times more likely to use LAPM as compared with those who had low knowledge (AOR = 7.8, 95% CI: 3.1, 18.3) [14, 19–23] in which participant’s knowledge was a major role in determining women intention to use PPIUCD. As observed from this and other similar studies, it is evident that knowledge serves as a major determinant of women intention to use PPIUCD.

## Conclusions

It was found only 34.9% women intended to use PPIUCD. Maternal age, maternal educational level, occupational status, and monthly income, knowledge and attitude were significantly associated with pregnant women intention to use PPIUCD. Since the proportion of pregnant mothers who intend to use PPIUCD is low every effort should be made to organize and implement community based information education and communication on postpartum intrauterine contraceptive device and involving pregnant women in family planning programs is essential. This could be achieved through the development and implementation of strategies that specifically target use of PPIUCD scale up program in the area.

## Data Availability

All data generated or analyzed during the study was included in this published article and its. additional information files. The raw data and materials are available and can be obtained from Addis Ababa University School of Nursing and Midwifery research and publication committee, Ethiopia and corresponding author is available on reasonable request.

## Abbreviations

ANC: Antenatal care
AOR: Adjusted odd ratio
CI: Confidence interval
COR: Crude odd ratio
CWC: Child welfare clinic
EDHS: Ethiopian demographic health survey
ETB: Ethiopian birr
HC: Health center
IUCD: Intra-uterine contraceptive device
LAPMs: Long acting and permanent method
MCHIP: Maternal and child health integrated program
MDG: Millennium development goal
MMR: Maternal mortality ratio
MoH: Ministry of health
NGO: Non-governmental Organization
PPIUCD: Post partum inta-uterine contraceptive device
SD: Standard deviation
SPSS: Statistical package for social sciences
WHO: World health Organization
